# Assessment of Setup Errors in Gynecological Malignancies Treated With Radiotherapy Using Onboard Imaging

**DOI:** 10.7759/cureus.37435

**Published:** 2023-04-11

**Authors:** Aparajeeta LNU, Piyush Kumar, Arvind Kumar Chauhan, Pavan Kumar, Jitendra Nigam, Silambarasan NS, Navitha S

**Affiliations:** 1 Department of Radiation Oncology, Shri Ram Murti Smarak Institute of Medical Sciences, Bareilly, IND

**Keywords:** ctv-ptv margins, kv imaging, immobilization, igrt, set up errors

## Abstract

Introduction

Radiotherapy plays a vital role in the management of gynecological malignancies. However, maintaining patient position poses a challenge during daily radiotherapy treatment of these patients. This study identifies and calculates setup errors in interfraction radiotherapy and optimum clinical target volume-planning target volume (CTV-PTV) margins in patients with gynecological malignancies.

Material and methods

A total of 38 patients with gynecological malignancies were included in the study. They were treated with a dose of 50 Gy in 25 fractions for five weeks, followed by brachytherapy. All patients were immobilized using a 4-point thermoplastic cast. Anteroposterior and lateral images were taken thrice weekly for five weeks. Setup verification was done using kilovoltage images obtained using Varian On-board Imager (Varian Medical System, Inc., Palo Alto, CA). Manual matching was done utilizing bony landmarks such as the widest portion of the pelvic brim, anterior border of S1 vertebrae, and pubic symphysis in the X, Y, and Z axes, respectively.

Results

A total of 1140 images were taken. The individual systematic errors ranged from -0.24 to 0.17 cm (LR), -0.15 to 0.19 cm (AP) and -0.36 to 0.29 cm (CC) while the individual random errors ranged from 0.04 to 0.36 cm (LR), 0.06 to 0.33 cm (AP) and 0.10 to 0.29 cm (CC). The calculated CTV-PTV margins in LR, AP and CC directions were 0.17, 0.18, and 0.25 cm (ICRU-62); 0.28, 0.31 and 0.47 cm in LR, AP and CC directions (Stroom's), and 0.32, 0.36 and 0.55 cm (Van Herk) respectively.

Conclusion

Based on this study, the calculated CTV-PTV margin is 6 mm in gynecological malignancies, and the present protocol of 7 mm of PTV margin is optimum.

## Introduction

External beam radiotherapy (EBRT) to the pelvis is a routine treatment for patients with gynecological malignancies. Radiation therapy involves various processes: positioning, immobilization, simulation, delineation, and treatment delivery. Many studies have proven that the positioning of patients for pelvic radiotherapy is relatively inaccurate and subject to setup variations that are probably greater than other sites in the body [1,2]. In addition, the motion of external skin marks relative to internal structures, the non-rigid nature of the area, patient rotation, and day-to-day variations in rectal and bladder filling, for instance, make the pelvis relatively challenging to set up accurately.

Based on the various literature results, a setup margin can be given for attaining precision in radiotherapy planning and providing accuracy in treatment delivery. In order to ensure optimal dose delivery and better outcome, it is essential to reduce setup errors during radiotherapy treatment. Portal imaging is essential for minimizing setup errors and defining adequate clinical target volume-planning target volume (CTV-PTV) margins.

Any discrepancy between the planned and actual treatment position is known as a setup error. These errors result from differences accumulated during the treatment planning process and recur during treatment sessions, which cause a shift in the cumulative dose distribution. There are two types of setup errors. Systematic errors are repeatable errors in the treatment technique and constantly occur until corrective actions are performed. In contrast, random errors are occasional, variable factors like patient movement during radiation treatment [3,4].

Accuracy and reproducibility of the patient's position are fundamental to the successful delivery of radiation therapy. However, uncertainty exists in radiotherapy due to setup errors resulting in a difference between planned and delivered doses [5]. The primary aim of the study is to assess the setup errors in gynecological malignancies treated with radiotherapy using onboard imaging

## Materials and methods

Approval from Shri Ram Murti Smarak Institute of Medical Sciences (IRB No:SRMSIMS/ECC/2019-20/127) was given at the outset.

This study included 38 patients with gynecological malignancies treated with image-guided radiation therapy (IGRT) in the Department of Radiation Oncology from November 2019 to April 2021.

Immobilization and simulation 

All patients were immobilized using a fixed four-point thermoplastic cast system (2.5 mm thickness pelvic cast, manufactured by Klarity). Patients underwent contrast-enhanced CT (CECT) scan radiotherapy planning (RTP). CECT whole abdomen scans were done with a flat table insert. CT images of the simulation were acquired with the patient in the supine and treatment position along with fiducial markers. Fiducial markers used were 2 mm in diameter with small lead balls placed on the pelvic cast. In addition, 3 mm slice thickness images were obtained. These images were transferred through Digital Imaging and Communications in Medicine (DICOM-CT) into the eclipse treatment planning system (version 8.6.17, Varian Medical System, Inc., Palo Alto, CA) [5]. 

Delineation of structures

Gross Tumor Volume (GTV)

GTV as seen on clinical examination and radiological imaging.

Clinical Target Volume (CTV)

CTV primary: Delineation of CTV primary (including GTV, uterus, vagina, bilateral parametrium). CTV nodal: Delineation of CTV nodal (including pelvic lymph nodes: common iliac, external iliac, internal iliac, obturator, and presacral). PTV: 7 mm isotropic expansion beyond CTV was taken per department protocol.

Organ At Risk (OARS)

Delineation of OARs was done per Radiation Therapy Oncology Group (RTOG) guidelines.

Bladder

Inferiorly starting from its base and superiorly to the dome.

*Rectum* 

Inferiorly starting from the lowest level of ischial tuberosities (right or left). Contouring ended superiorly till the rectum lost its round shape in the axial plane and connected anteriorly with the sigmoid.

*Bowel Bag* 

Inferiorly from the most inferior small or large bowel loop, or above the rectum or anorectum, whichever is most inferior. Abdominal contents were contoured, excluding muscle and bones. Contouring was stopped superiorly 1 cm above PTV.

Bone Marrow

This comprised the whole pelvic bone, lumbar spine, and bilateral proximal femur.

Left and Right Femur

Cranially first section of the femoral head and caudally up to the lesser trochanter.

*Dose Prescription* 

All patients were delivered standard radiotherapy at a dose of 50 Gy in 25 fractions over five weeks along with concurrent Cisplatin at 35 mg/m2 i/v infusion every week.

Portal imaging and displacement measurements

After patient positioning, an orthogonal pair (anteroposterior and lateral) of portal images were acquired using an on-board kV imager on a linear accelerator. Reproducible bony landmarks were defined for evaluating patient setup errors as per the recommendations by the Royal College of Radiologists. Pelvic brim for X (left to right) displacements and pubic symphysis for Z (superior to inferior) displacements were taken as the bony landmarks in anterior-posterior portal images. The anterior border of the S1 vertebra for Y (anterior to posterior) displacements was identified as a bony landmark in lateral portal images [5]. For the X axis, displacement to the left was taken as positive, while displacement to the right was taken as negative. In the Z axis, displacement in the superior direction was taken as positive and displacement in the inferior direction as negative. In the Y axis, anterior displacement was taken as negative and posterior displacement was taken as positive.

Calculation of systematic and random errors

Individual and population-based systematic and random errors were calculated along the X, Y, and Z directions. These were calculated according to the report by the Royal College of Radiologists. The report describes the individual mean setup error M_individual_ as the mean setup error for an individual patient. The overall population mean setup error M_pop_ is defined as the overall mean for the analyzed patient group. The population systematic error Σ^2^ _set-up_ is defined as the SD of the individual mean setup error about the overall mean M_pop_. The individual random (daily) setup error σ^2^_individual_ is defined as the SD of the setup error around the corresponding mean individual value M_individual_. Finally, the population random error σ_set-up_ is defined as the mean of all setup individual random errors σ_individual_ [5].

Calculation of CTV-PTV margin

After the calculation of systematic and random error values, population-based CTV-PTV margins were calculated for all patients using the International Commission on Radiation Units and Measurements (ICRU) Report 62, Stroom’s (2 ∑ + 0.7 σ), and Van Herk’s formulae (2.5 ∑ + 0.7 σ) [6,7].

Treatment execution and verification

Treatment was delivered with 6MV and 15 MV X-rays by a linear accelerator. All patients receiving pelvic radiation therapy underwent kilovoltage portal (kVp) imaging using On-Board Imager on a regular basis on the linear accelerator. In each patient, kV images (AP and lateral) were taken three times a week (Monday, Wednesday, and Friday), and a cone beam CT scan (CBCT) image was taken once every week (Tuesday) for a total of five weeks. Total kV images for each patient were 15 (AP and lateral). A single observer did all observations to remove inter-observer bias.

## Results

Displacement measurements

The displacements of 38 patients were calculated in 1140 images, and assessment was done in X (left to right), Y (anterior to posterior), and Z (superior to inferior) directions.

**Figure 1:**
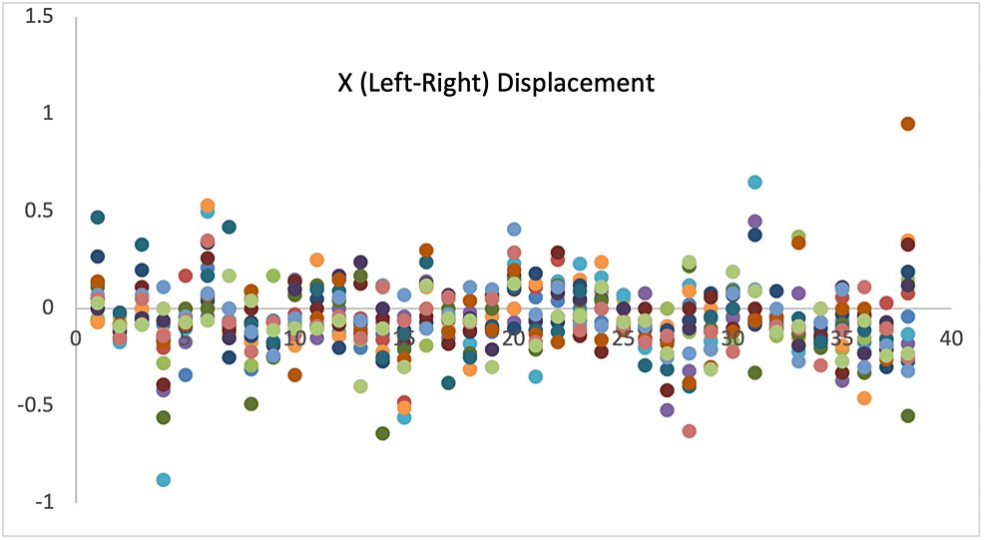
Displacement in X-axis.

**Figure 2:**
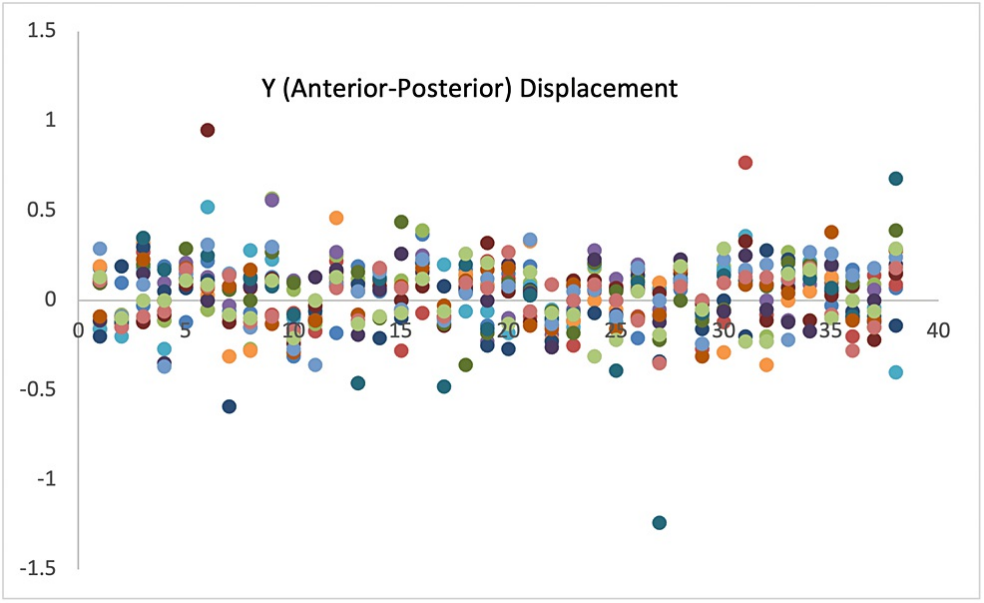
Displacement in Y-axis.

**Figure 3:**
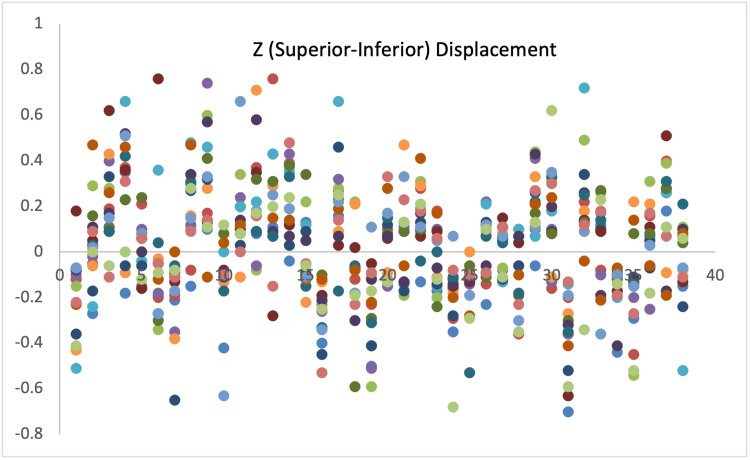
Displacement in Z-axis.

Displacement measurements of all 38 patients are demonstrated with scatter diagrams (Figures 1-3).

Errors

Mean Individual Error

Mean individual errors ranged from a minimum of -0.36 cm on Z-axis to a maximum of 0.29 cm on Z-axis (Table 1).

**Table 1:** Mean individual errors of patients on X, Y and Z axis.

S.No.	X coordinate (cm)	Y coordinate (cm)	Z coordinate (cm)
M_1_	0.07	0.03	-0.18
M_2_	-0.11	-0.08	0.03
M_3_	0.02	0.13	0.20
M_4_	-0.23	-0.06	0.27
M_5_	-0.07	0.13	0.01
M_6_	0.17	0.18	-0.06
M_7_	-0.03	-0.03	-0.18
M_8_	-0.12	-0.01	0.21
M_9_	-0.14	0.17	0.29
M_10_	-0.04	-0.14	-0.11
M_11_	-0.03	-0.09	0.16
M_12_	0.00	0.14	0.22
M_13_	-0.07	-0.05	0.19
M_14_	-0.09	0.07	0.24
M_15_	-0.24	0.03	-0.00
M_16_	0.05	0.19	-0.26
M_17_	-0.07	-0.08	0.21
M_18_	-0.10	0.08	-0.11
M_19_	-0.06	0.02	-0.26
M_20_	0.12	0.04	0.05
M_21_	-0.07	0.07	0.06
M_22_	0.06	-0.11	0.16
M_23_	0.01	-0.05	-0.04
M_24_	0.02	0.09	-0.20
M_25_	-0.04	-0.05	-0.16
M_26_	-0.11	0.05	0.03
M_27_	-0.20	-0.15	0.02
M_28_	-0.11	0.13	-0.08
M_29_	-0.12	-0.11	0.22
M_30_	-0.01	0.05	0.17
M_31_	0.07	0.17	-0.36
M_32_	-0.07	-0.03	0.20
M_33_	-0.06	0.06	0.05
M_34_	-0.12	0.13	-0.17
M_35_	-0.10	0.11	-0.18
M_36_	-0.15	-0.03	0.04
M_37_	-0.16	-0.05	0.18
M_38_	0.01	0.17	-0.05

Systematic and Random Errors

The calculated population systematic errors were 0.09 cm on the X axis, 0.10 cm on the Y axis, and 0.17 cm on the Z axis, and the calculated population random errors were 0.14 cm on the X axis, 0.15 cm on the Y axis, and 0.18 cm on the Z axis (Table 2).

**Table 2:** Random and systematic errors on X, Y, and Z axis.

	Random error (RE) (cm)	Systematic error (SE) (cm)
X	0.14	0.09
Y	0.15	0.10
Z	0.18	0.17

CTV-PTV Margins

Population-based CTV-PTV margins were calculated for all patients using the ICRU Report 62, Stroom's, and Van Herk's formulae [5]. Using the ICRU recommendation, the CTV-PTV margin in LR, AP, and CC directions were 0.17 cm, 0.18 cm, and 0.25 cm, respectively. The corresponding values were 0.28 cm, 0.31 cm, and 0.47 cm according to Stroom's formula and 0.32 cm, 0.36 cm, and 0.55 cm according to Van Herk's formula (Table 3).

**Table 3:** Calculated CTV-PTV margins. CTV-PTV: Clinical target volume-planning target volume; ICRU: International Commission on Radiation Units and Measurements.

ICRU 62			Stroom’s			Van Herk		
LR (cm)	AP (cm)	CC (cm)	LR (cm)	AP (cm)	CC (cm)	LR (cm)	AP (cm)	CC (cm)
0.17	0.18	0.25	0.28	0.31	0.47	0.32	0.36	0.55

## Discussion

There are two types of setup errors: random and systematic errors.

The systematic error is the deviation that occurs in the same direction and is of a similar magnitude for each fraction throughout the treatment course. For example, this could be due to the target delineation error or change in target position and shape between delineation and treatment (tumor regression, bladder and bowel changes) [5].

The random error is the deviation that varies in direction and magnitude for each delivery fraction. This may be due to patient setup error changes in target position and shape between fractions and during fractions (like breathing). In addition, random errors are influenced by immobilization technique, patient comfort, and departmental protocols [5].

Our study attempts to calculate the setup errors in all three axes and the final CTV-PTV margins. Daily setup was recorded in all three planes, i.e., X, Y, and Z. kV images were matched based on bony landmarks. The setup errors identified were used to calculate the systematic and random errors. Varian offline data was used for documenting the shift in all the planes for daily fractions in all the patients. The clinical shifts were used for correcting the setup errors, and they were read and run by the same person. The mean deviation and SD of the individual and population were calculated. Based on the available values, the calculation of the CTV-PTV margin was done in the X, Y, and Z axis based on Stroom, Van Herk, and ICRU 62 formulae.

In their study on 22 patients treated with pelvic radiotherapy, Noghreiyan VV et al. evaluated a total of 182 portal images. Population's random (σ) and systematic (Σ) errors were calculated based on the portal images in three directions (X, Y, and Z). The systematic setup errors for patients ranged between 2.36 and 4.99 mm, while the random errors ranged between 1.51 and 2.74 mm. The setup margin for CTV to PTV was in the range of 2.8-5.7 mm, 5.7-11.9 mm, and 6.9-14.4 mm in the X, Y, and Z directions, respectively [8].

In our study, the decrease in setup margins could possibly be due to the increased number of images taken in our study (thrice weekly) along with CBCT imaging. The advantage of CBCT imaging is that multiple slices in different planes and soft tissue resolution allow for more precise correction of setup errors.

In our study, CTV-PTV margins did not exceed 0.55 cm, possibly because of rigid immobilization devices, which reduced the setup errors in all three directions. Using a 4-point thermoplastic cast for all the patients, the calculated CTV-PTV margins according to the Van Herk formula were 0.32 cm, 0.36 cm, and 0.55 cm in LR, AP, and CC directions, respectively. While analyzing the setup errors, it was observed that maximum displacement occurred in the craniocaudal direction. 

In a study by Murrell DH et al. done on 20 patients with cervical and endometrial malignancies, daily CBCT for each was registered in four dimensions to the planning CT. The bony landmarks chosen were the sacrum and pubic symphysis in the anterior-posterior (A-P) direction, lower lumbar vertebrae, and ischial tuberosity in the superior-inferior (S-I) direction, and the femoral heads laterally [9]. The median shift between IGRT methods was 2 mm, 1 mm, and 1 mm in the anterior-posterior, superior-inferior, and lateral directions, respectively. Maximum deviations were observed in the A-P direction [9]. The possible reason for the reduction in setup margin lies in the difference in imaging protocol and CBCT imaging (CBCT images were taken once a week). Also, they used different bony landmarks for recording the setup errors in craniocaudal, anteroposterior, and mediolateral directions. This led to more accurate daily reproduction of treatment and a reduction in setup margins. We need to review the pros and cons of daily verification by CBCT versus the daily radiation hazard of CBCT.

In our institute, a similar study was done five years back by Kumar P et al. on 21 patients with carcinoma cervix. Patients were immobilized using full-body Vaclok cushions. A total of 242 images were evaluated. The individual systematic errors calculated were -6.6 to 4.9 mm on the X axis, -4.9 to 3.5 mm on Y-axis, and -6.3 to 6.5 mm on Z-axis. In contrast, individual random errors ranged from 0.5 to 8.3 mm, 0.7 to 5.2 mm, and 1.1 to 4.6 mm on the X, Y, and Z axis, respectively. CTV-PTV margins were 7.9 mm, 7.0 mm, and 9.1 mm on the X, Y, and Z axis, respectively. They found that safety margins of 1 cm would be adequate for all the patients [5]. In our study, utilizing 4-point thermoplastic casts for immobilization and daily kV imaging for verification, we got CTV-PTV margins of less than 6 mm. As a result, the systematic and random errors obtained were also lesser than their study.

In our institute, we initially followed 1 cm of PTV margin [5]. With the introduction of rigid immobilization devices (thermoplastic casts) and better imaging techniques (IGRT), the margins were gradually reduced to 7 mm (symmetric margins). Our study calculated the maximum setup margin as 6 mm, thus inferring that a further reduction of 1 mm in setup margin could be made in the near future, eventually leading to more normal tissue sparing and less toxicity.

Our study has two limitations. First, rotational errors were not taken into account because of the lack of six degrees of freedom couch which could be incorporated in further studies as per the availability. Also, it is to be noted that the organ motion was not accounted for in our study. Hence, further studies are required in this direction.

We assessed the various setup uncertainties in each direction to generate our own CTV-PTV margins. Our study showed that the measured setup uncertainties were lesser than the estimated errors, and the institutional protocol of a 7 mm margin is adequate. These setup errors cannot be generalized because of variation in the procedures used, which include the type of immobilization, infrastructure present, and type of imaging system available. PTV margins can only be validated after doing a small research on setup errors in the department. In our institute, patients were treated five times a week, but the protocol was based on three times kV images and one-time CBCT due to research protocol. Further reduction of setup margins is justified by daily patient positioning, localization, and correction before treatment. The establishment of online correction protocols would further help improve treatment positioning accuracy [10]. An adequate immobilization system is a key factor in determining setup errors, and the appropriateness of thermoplastic cast needs to be ensured. The expertise of treatment team members and quality assurance are also essential. While determining the institutional protocol for PTV margins other than imaging protocols and immobilization, these numerous factors also need to be taken into account.

## Conclusions

It is helpful to audit the PTV margins practiced in the department at regular intervals and try to decrease the setup errors by using stringent methodology protocols for imaging. Present PTV margin of 7 mm practiced in our department was found to be optimum. However, every institution needs to define its own PTV margins after research work, depending on the type of immobilization used and the available imaging facilities.
